# Posterior Lumbar Arthrodesis Using a Stand-Alone Hydrophilic Synthetic Bone Graft in the Setting of Postoperative Osteomyelitis/Discitis: A Case Report

**DOI:** 10.7759/cureus.116061

**Published:** 2026-09-10

**Authors:** Micah Smith

**Affiliations:** 1 Spine Surgery, Ortho Northeast (ONE), Fort Wayne, USA

**Keywords:** bone void filling, corpectomy, discitis, osteoflo hydrofiber, osteomyelitis, posterior lumbar fusion, revision spine surgery, spinal arthrodesis, spondylodiscitis, synthetic bone graft

## Abstract

This single-patient case report describes an all-posterior salvage reconstruction of multilevel lumbar osteomyelitis using a stand-alone hydrophilic synthetic bone graft (HPSBG; OsteoFlo® HydroFiber™, SurGenTec, LLC, Boca Raton, FL) packed transpedicularly as the sole intervertebral graft material. Postoperative osteomyelitis with progressive vertebral body destruction commonly involves combined anterior-posterior reconstruction with corpectomy and structural cage placement, which is technically demanding and morbid in patients with poor bone quality and adjacent prior fractures. Hydrophilic synthetic bone grafts engineered to support fusion in compromised environments may permit less invasive salvage strategies.

A 59-year-old woman with persistent L1-L2 osteomyelitis after a prior L2-L5 fusion for an L3 burst fracture, complicated by osteoporosis and low vitamin D levels (25-hydroxyvitamin D 15.6 ng/mL), underwent posterior-only salvage reconstruction. Through an all-posterior approach, transpedicular partial corpectomies of L1 and L2 were performed through the existing erosive defects. Intraoperative cultures grew *Pseudomonas aeruginosa*. The void was packed with HPSBG as the sole intervertebral graft material, with no anterior cage. Long-segment T10-pelvis fixation with a quad-rod construct provided posterior stabilisation. Computed tomography (CT) at eight months postoperatively demonstrated bridging bone through the L1-L2 defect, consistent with early anterior column consolidation, and repeat CT at 12 months showed bridging trabecular bone across the defect with continuity to the host endplates. Patient-reported outcomes improved at 12 months, and the patient returned to her preoperative activities. This case suggests that a stand-alone hydrophilic synthetic graft may support anterior column consolidation when used without an interbody cage in an infected, biologically hostile environment, potentially reducing the need for morbid anterior reconstruction in selected salvage cases. Larger comparative studies are warranted.

## Introduction

Lumbar osteomyelitis and discitis are typically managed with bracing and intravenous antibiotics, with surgical intervention reserved for neurological deficits, intraspinal abscess, mechanical instability, or failure of medical management [1, 2]. Postoperative osteomyelitis arising in the presence of existing instrumentation represents a particularly challenging clinical scenario. When infection progresses despite debridement and targeted antibiotic therapy, erosive vertebral body destruction can produce instability that mandates salvage reconstruction [1, 2]. These operations are characteristically morbid, with significant rates of non-union and re-operation, and are further complicated by patient comorbidities such as osteoporosis, vitamin D deficiency, and prior adjacent fractures [1, 2].

The traditional reconstructive approach in this setting is combined anterior-posterior surgery, with corpectomy of the involved levels performed anteriorly, debridement, structural cage placement, and supplementary posterior instrumentation. Anterior approaches add operative time, blood loss, and access-related morbidity, and may be poorly tolerated in deconditioned patients. However, combined anterior-posterior surgery is not universally required; posterior-only debridement and instrumented reconstruction is well established for many cases of spondylodiscitis, and the posterior approach can itself restore the anterior column. Bone graft options for infected, biologically compromised fields are limited: iliac crest autograft adds donor site morbidity, vascularized struts increase complexity and operative duration, and allograft tissues carry donor-to-donor variability. Next-generation hydrophilic synthetic bone grafts have been engineered to promote vascularisation, osteoblast activity, and early bony ingrowth in compromised environments [3-6, 7-10]. The graft used in this case, OsteoFlo® HydroFiber™ (SurGenTec, LLC, Boca Raton, FL; hereafter HPSBG), is a hydrophilic synthetic bone graft in which porous particles of silicate-substituted carbonate apatite are suspended within a web-interlaced synthetic fibre scaffold, FDA-cleared as a bone void filler that may be used stand-alone in appropriate osseous defects, including defects created by cysts, tumours, and osteomyelitis. It was selected less for its osteoconductivity, which it shares with the synthetic class, than for its cohesion in a wet, non-contained defect: in comparative in vitro benchmarking within a hydrated interbody model, 10 commercially available comparator grafts all dissociated on inversion within 48 hours, whereas this scaffold retained cohesion [11]. That study was manufacturer-funded, single-replicate, and static in vitro. Where a graft is delivered transpedicularly into an irregular cavity without a cage, resistance to dissociation is a governing consideration.

I report a salvage case in which HPSBG was packed transpedicularly into a large L1-L2 erosive defect, without any anterior cage or anterior instrumentation. Early anterior column consolidation was observed at eight months, with progression on repeat CT at 12 months. Stand-alone use of this graft in multilevel lumbar osteomyelitis has been reported previously, with the graft contained within a titanium corpectomy cage that supplied anterior column support [12]. To my knowledge, however, this is the first reported case in which HPSBG served as the sole intervertebral material, without any interbody or corpectomy cage, for all-posterior anterior column reconstruction in lumbar osteomyelitis.

## Case presentation

**Figure 1:**
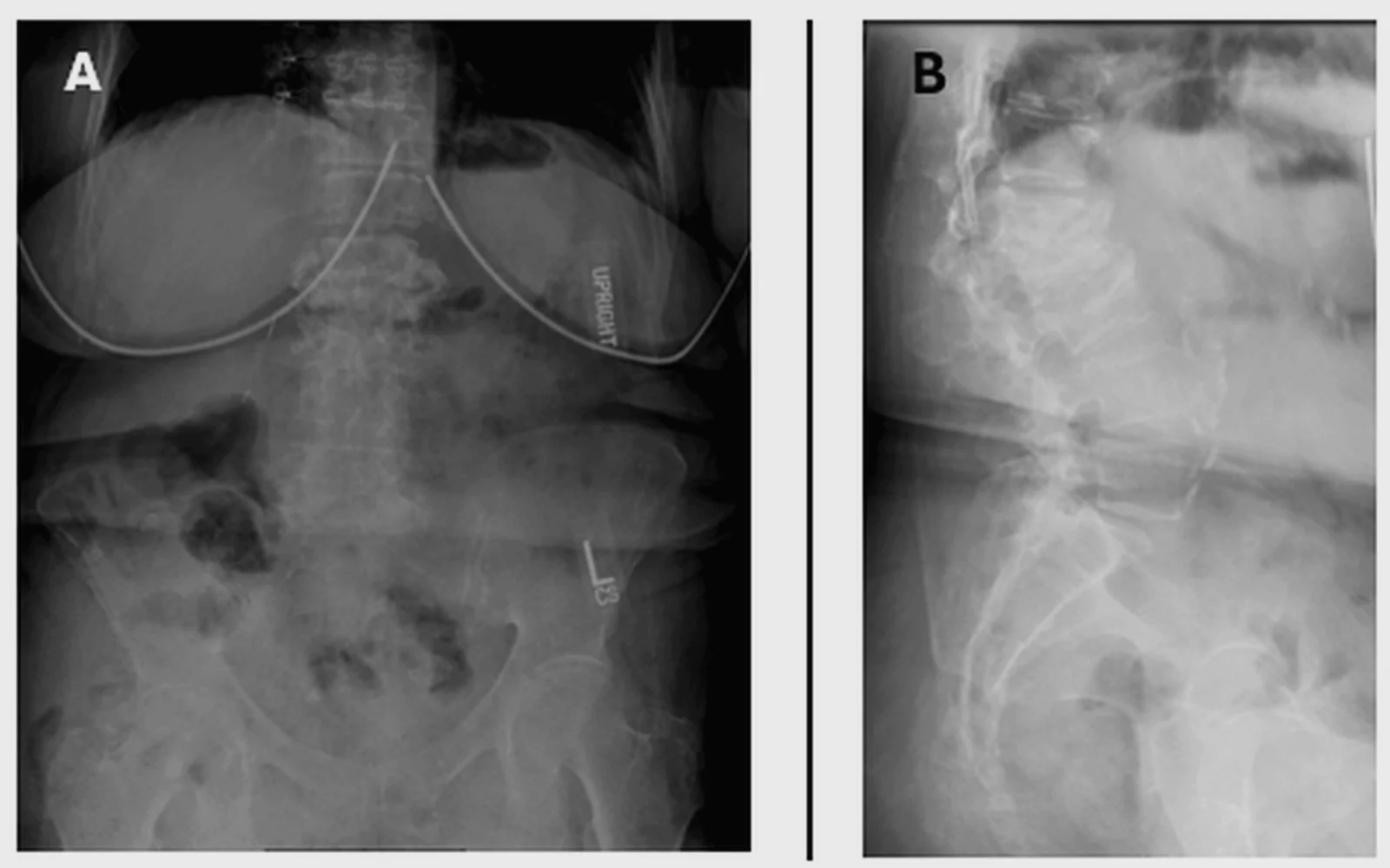
(A) Preoperative standing anteroposterior radiograph and (B) lateral radiograph of the lumbar spine after removal of the prior L2-L5 instrumentation, demonstrating absence of hardware and progressive kyphotic alignment.

**Figure 2:**
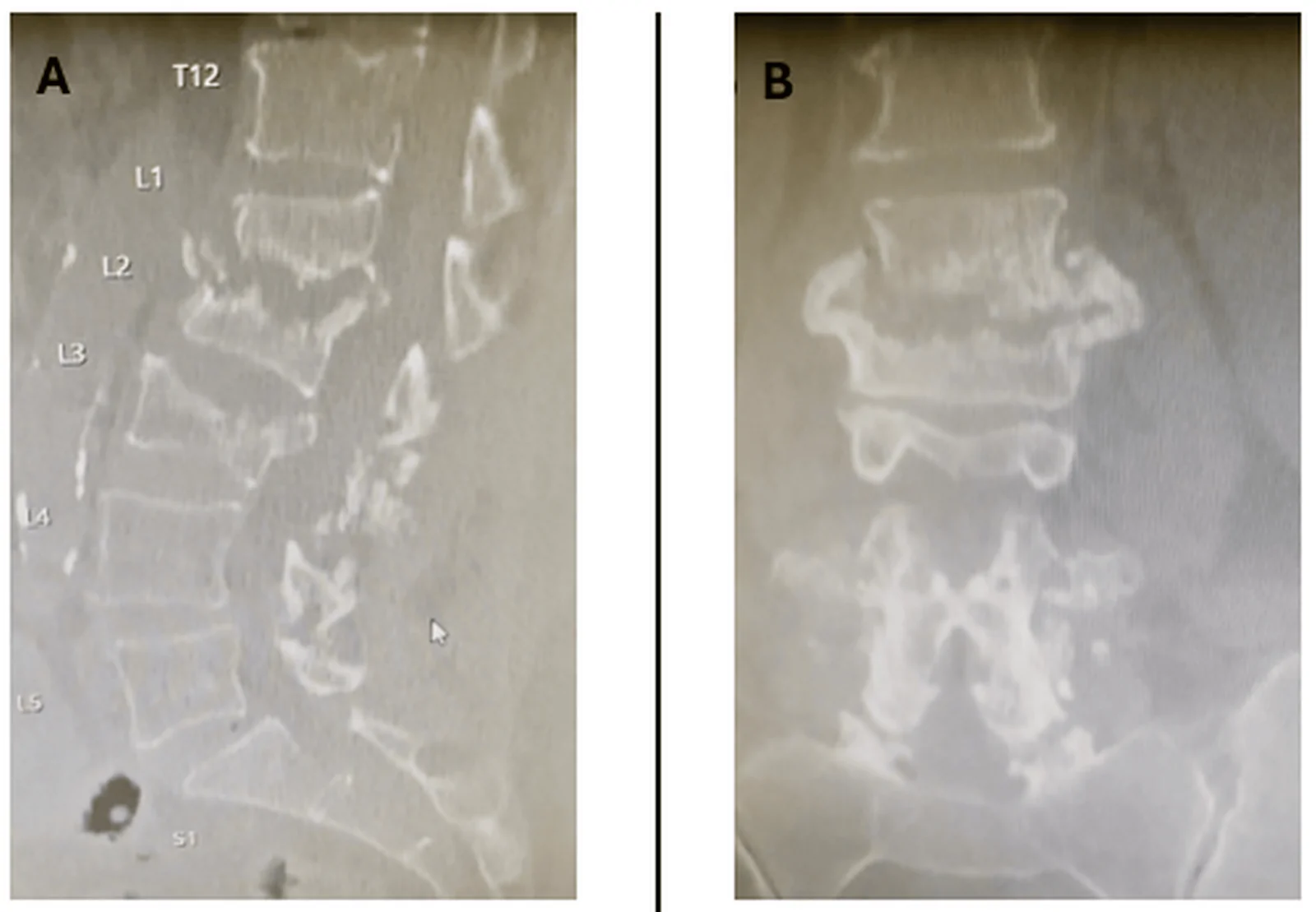
(A) Preoperative sagittal CT of the lumbar spine demonstrating extensive bony destruction at L1 and L2 with endplate irregularity, consistent with persistent osteomyelitis and discitis, (B) Preoperative coronal CT of the lumbar spine confirming extensive bony destruction of the same segment.

**Figure 3:**
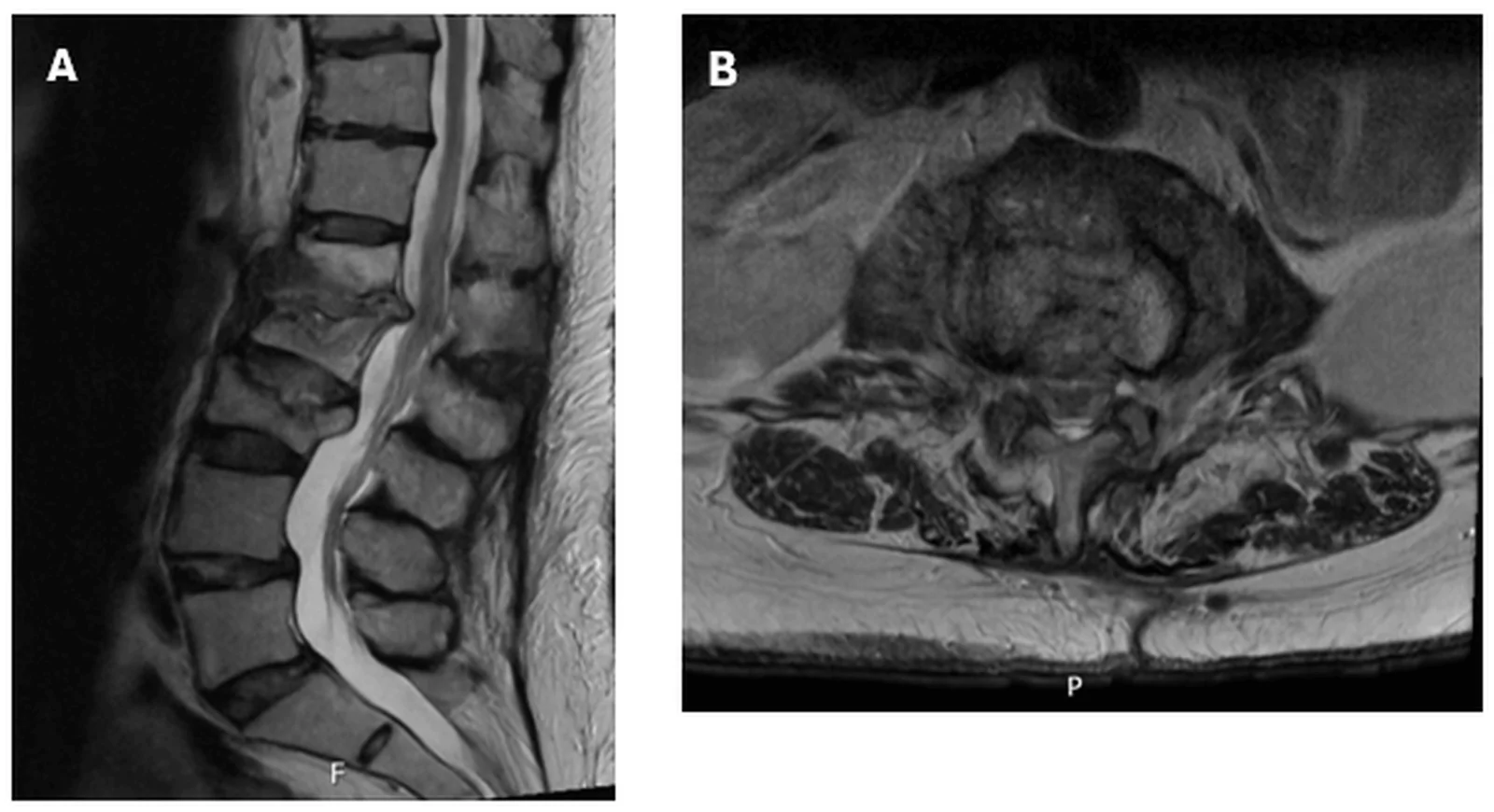
(A) Preoperative sagittal MRI demonstrating progressive height loss of L1 and L2 with inflammatory and destructive changes at L1-L2 consistent with persistent osteomyelitis and discitis; abnormal signal at the L3 superior endplate, of uncertain significance (granulation tissue versus infection), is also visible; and (B) Axial MRI demonstrating the inflammatory and destructive changes at L1-L2 consistent with persistent osteomyelitis and discitis.

The patient was a 59-year-old woman who had undergone L2-L5 posterior instrumented fusion for an L3 burst fracture at an outside institution and presented six months after removal of that instrumentation. Her postoperative course had been complicated by deep infection, for which she had previously undergone excisional debridement and intravenous antibiotic therapy. Despite this, she failed antibiotic management and required a second debridement with removal of instrumentation. She subsequently completed six weeks of intravenous daptomycin and cefepime as directed at the treating institution. She subsequently presented with intractable back pain and bilateral lower extremity radicular pain in the setting of stenosis and large erosive lesions of the L1 and L2 vertebral bodies, consistent with persistent osteomyelitis and discitis. Her clinical picture was further complicated by osteoporosis and a low 25-hydroxyvitamin D level of 15.6 ng/mL, below the commonly accepted adequacy threshold of ≥20 ng/mL and consistent with vitamin D insufficiency/deficiency. At presentation, C-reactive protein was <3 mg/L and erythrocyte sedimentation rate was 41 mm/hr. She was started on teriparatide (Forteo®, Eli Lilly and Company, Indianapolis, IN) for several months prior to surgical stabilisation. Standing anteroposterior and lateral radiographs after instrumentation removal demonstrated the absence of hardware and progressive kyphotic alignment (Figure 1). Sagittal computed tomography (CT) confirmed extensive bony destruction at L1 and L2 with endplate irregularity (Figure 2). Sagittal and axial magnetic resonance imaging (MRI) demonstrated progressive height loss of L1 and L2 with continued evidence of osteodiscitis and vertebral osteomyelitis, contrast-enhancing tissue, and enhancement of the L3 superior endplate of uncertain significance, either granulation tissue or infection (Figure 3). Table 1 summarizes the clinical timeline from the index fusion through 12-month follow-up.

Proximal fixation was extended to T10 to obtain three levels of segmental purchase above the infected, eroded vertebrae, given the severe anterior destruction and poor bone quality. Distal fixation was carried to the pelvis rather than stopping at L5 because of a pre-existing L5-S1 anterolisthesis that placed a distal L5 foundation at high risk of failure and distal junctional instability; to maximise distal purchase, four points of pelvic fixation were obtained. Anterior column support with corpectomy of L1 and L2 was considered. Because the prior L3 fracture left the endplates without normal contour to support a corpectomy cage, an L3 corpectomy would also have been required, ultimately necessitating anterior fusion from T12 to L4. Given the potential morbidity of that procedure, a robust posterior stabilisation was planned instead. Because corpectomy at L1-L3 remained possible and L1-L2 were actively infected, no screws were placed at L1, L2, or L3. Cement augmentation was avoided given the active infection and the concern for seeding osteomyelitis at additional levels.

The patient underwent a posterior-only reconstruction with long-segment T10-pelvis fixation using bilateral S1 alar-iliac and S2 alar-iliac screws. Through a standard open posterior midline approach and under intraoperative navigation, navigated shavers and straight and angled curettes were passed through what remained of the L2 pedicle to debride the L1 and L2 vertebral bodies, performing partial corpectomies posteriorly through the existing large erosive defects. A long angled curette passed through the L2 pedicle allowed debridement to extend cranially into the L1 vertebral body, and navigation facilitated safe but aggressive debridement of the infected vertebral bodies. The intervertebral and vertebral void was packed with HPSBG, utilising a graft funnel and pusher transpedicularly into the L1-L2 disc space and bony defect to serve as the sole intervertebral graft material. The graft was hydrated with sterile saline per manufacturer instructions for use. Calcium-sulfate beads mixed with antibiotics were utilised to provide local release/control. No anterior cage or anterior instrumentation was placed. Intraoperative cultures grew Pseudomonas aeruginosa, susceptible to meropenem and intermediate to ceftazidime and cefepime; the isolate was not multidrug-resistant. The infectious disease service directed six weeks of intravenous meropenem postoperatively. A quad-rod construct was used over the region of greatest bone loss to augment posterior stability.

**Figure 4:**
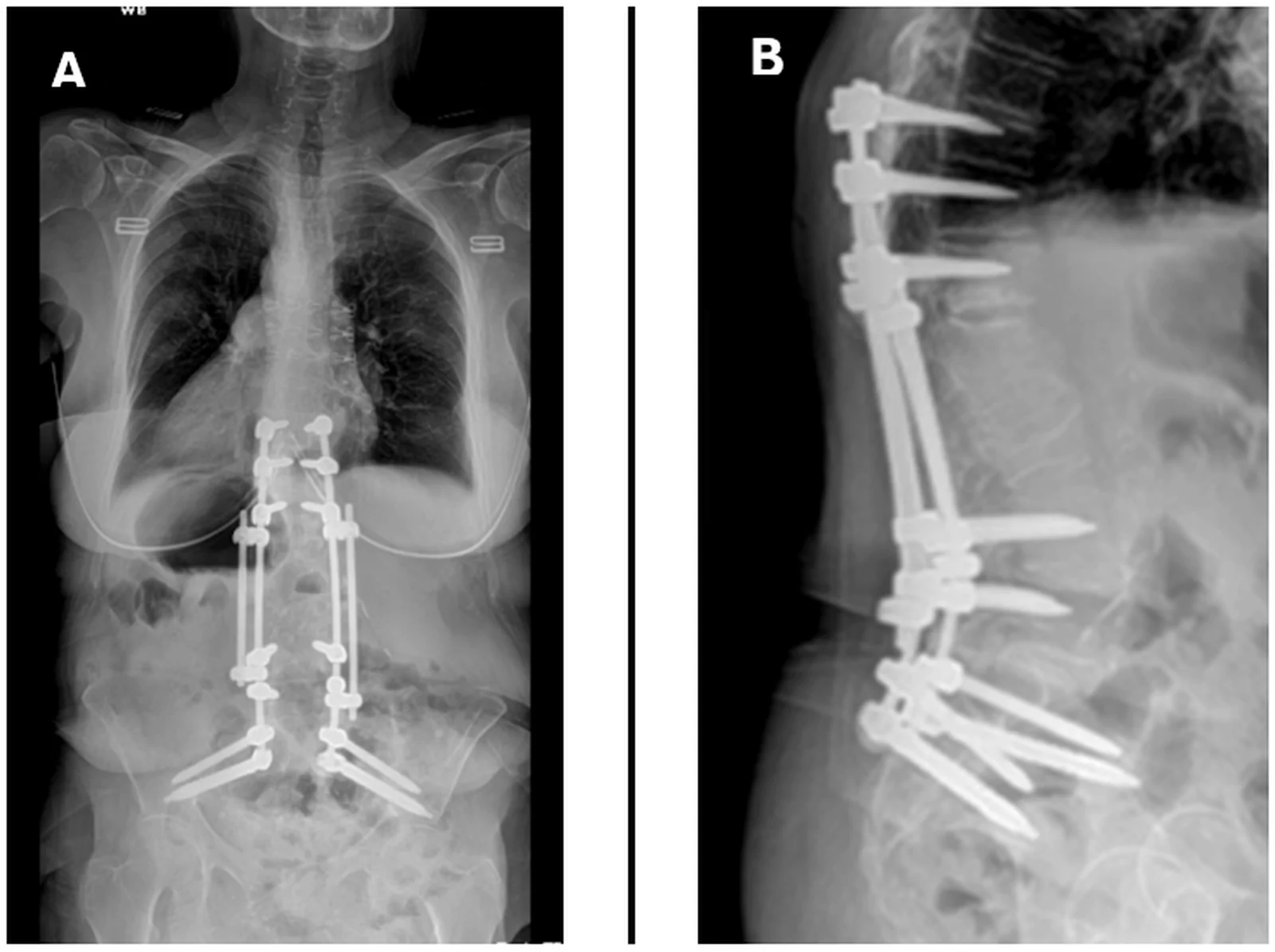
(A) Postoperative standing anteroposterior radiograph and (B) lateral radiograph demonstrating the long-segment T10–pelvis quad-rod construct and improved sagittal alignment.

**Figure 5:**
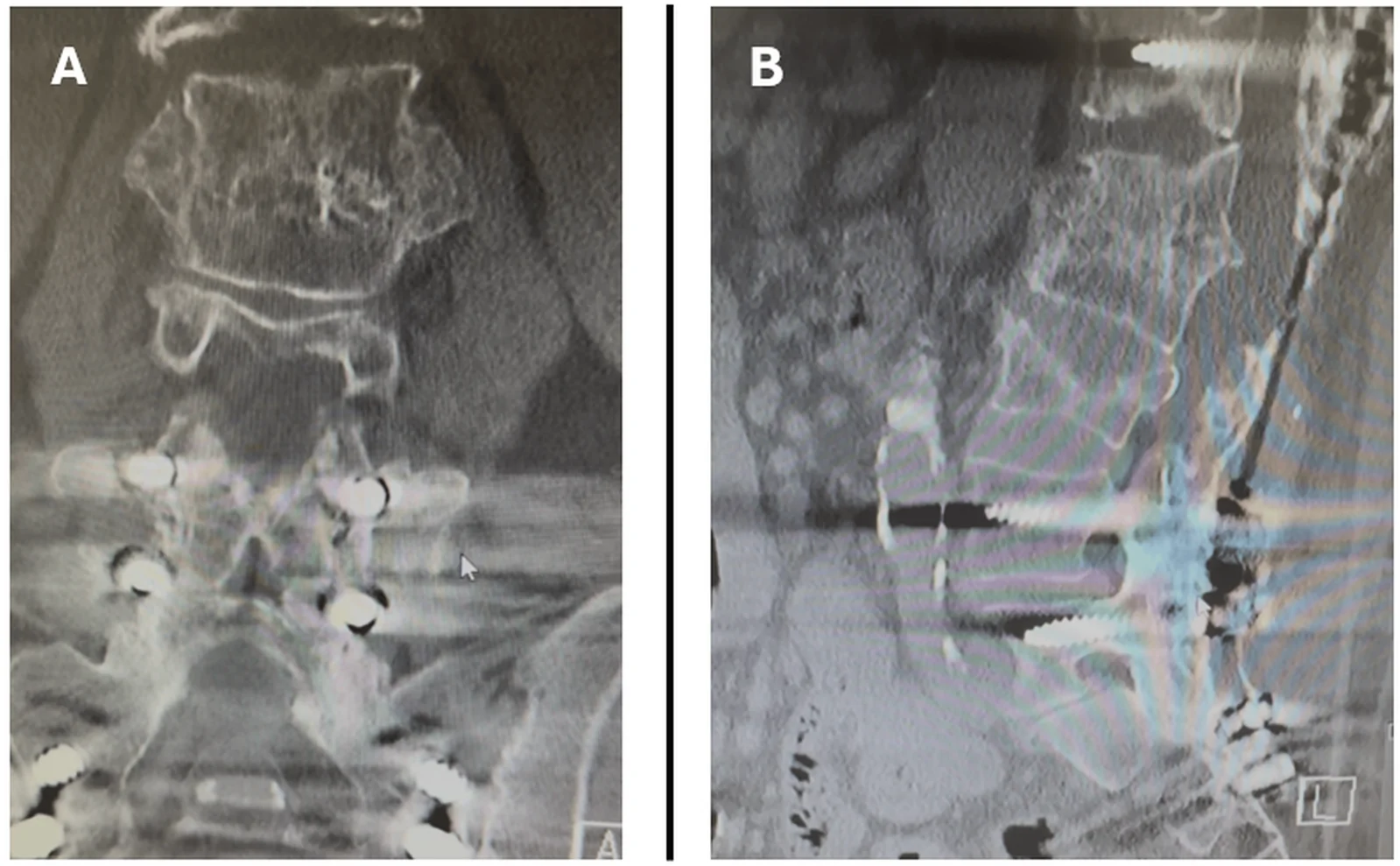
Postoperative CT imaging at eight months demonstrating bridging bone and early anterior column consolidation through the L1-L2 defect. (A) Coronal and (B) sagittal reconstructions show consolidated bone graft through the transpedicularly packed void in the absence of any anterior cage.

**Figure 6:**
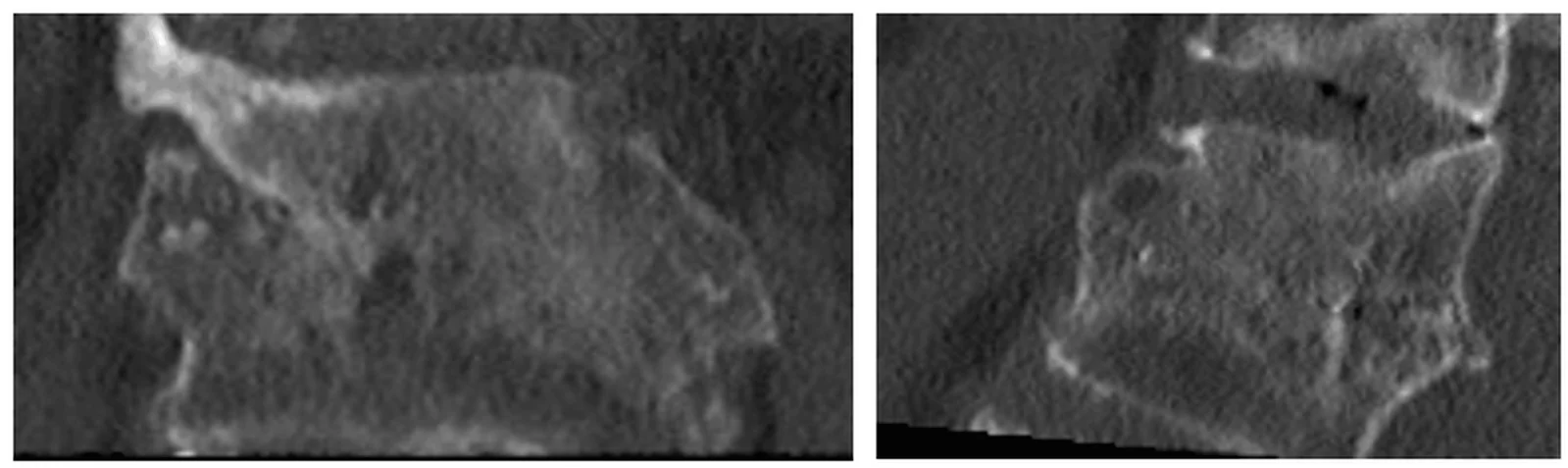
Postoperative CT at 12 months demonstrating bridging trabecular bone across the L1-L2 defect with continuity to the host endplates and no lucency, showing progression from the early consolidation seen at 8 months (Figure 5).

Postoperative AP and lateral standing radiographs demonstrated improved sagittal alignment, with lumbar lordosis increasing from 21° preoperatively to 40° postoperatively and a postoperative PI-LL mismatch of 13°, and showed the long-segment T10-pelvis quad-rod construct (Figure 4). CT imaging at eight months postoperatively demonstrated bridging bone through the L1-L2 defect with early anterior column consolidation (Figure 5). Postoperative imaging showed some proximal junctional change at the upper instrumented level, which was asymptomatic and did not require revision through 12 months. Repeat CT at 12 months showed bridging trabecular bone across the defect with continuity to the host endplates and no lucency (Figure 6).

Patient-reported outcomes, collected immediately before surgery and at 12 months, improved across all instruments: Oswestry Disability Index 67 to 38, PROMIS Physical Function T-score 24 to 36, back pain visual analogue scale 10 to 7, and leg pain visual analogue scale 8 to 0. Through 12 months, there was no clinical, laboratory, or radiographic evidence of recurrent infection, and no graft-associated infection or other graft-related complication was observed. The patient rated her satisfaction with surgery as 10 of 10 and had returned to her preoperative recreational activities, including gardening. The clinical course from the index fusion through 12-month follow-up is summarised in Table 1.

**Table 1:** Clinical timeline from index surgery to 12-month follow-up. (Patient: 59-year-old female with osteoporosis and vitamin D deficiency.) CRP: C-reactive protein; ESR: erythrocyte sedimentation rate; CT: computed tomography; MRI: magnetic resonance imaging; PROMIS: Patient-Reported Outcome Measurement Information System

Time point	Event	Findings and management
Index surgery (outside institution)	L2-L5 posterior instrumented fusion for an L3 burst fracture	-
Postoperative course	Postoperative infection with L1-L2 osteomyelitis and discitis	Irrigation and debridement with intravenous antibiotic therapy.
Persistent infection	Infection continued despite debridement and intravenous antibiotics	Removal of all instrumentation by the treating surgeon, followed by 6 weeks of intravenous daptomycin and cefepime as directed at the treating institution.
Presentation for salvage (6 months after instrumentation removal)	Continued and worsening back and radicular pain; bilateral lower-extremity radiculopathy and stenosis	Comorbidities: osteoporosis; 25-hydroxyvitamin D 15.6 ng/mL; teriparatide started for several months preoperatively. Laboratory: CRP <3 mg/L; ESR 41 mm/hr. Imaging: standing radiographs (absence of hardware, progressive kyphotic alignment; Figure 1); CT (extensive L1-L2 destruction with endplate irregularity; Figure 2); MRI (progressive height loss of L1 and L2, continued osteodiscitis and vertebral osteomyelitis, contrast-enhancing tissue, and enhancement of the L3 superior endplate of uncertain significance; Figure 3).
Salvage surgery	All-posterior reconstruction	Long-segment T10-pelvis fixation with bilateral S1 alar-iliac and S2 alar-iliac screws; quad-rod construct spanning T12-L4/L5 over the region of greatest bone loss; no screws at L1-L3; no cement augmentation. Navigated shavers and straight and angled curettes were used through the remaining L2 pedicle to perform transpedicular partial corpectomies of L1 and L2. Hydrophilic synthetic bone graft (HPSBG) hydrated with sterile saline and packed transpedicularly via graft funnel and pusher as the sole intervertebral material; antibiotic-loaded calcium-sulfate beads for local release; no anterior cage or anterior instrumentation. Postoperative radiographs showed lumbar lordosis improved from 21° to 40° with a PI-LL mismatch of 13° (Figure 4)
Salvage microbiology	Intraoperative cultures	Pseudomonas aeruginosa, susceptible to meropenem and intermediate to ceftazidime and cefepime; not multidrug-resistant. Infectious disease service directed 6 weeks of intravenous meropenem.
8 months postoperatively	Radiographic follow-up	CT demonstrated bridging bone through the L1-L2 defect with early anterior column consolidation (Figure 5).
12 months postoperatively	Radiographic follow-up	Repeat CT demonstrated bridging trabecular bone across the L1-L2 defect with continuity to the host endplates and no lucency (Figure 6).
12 months postoperatively	Clinical and functional follow-up	No evidence of recurrent infection. Patient-reported outcomes improved from before surgery to 12 months: ODI 67→38; PROMIS Physical Function T-score 24→36; back pain VAS 10→7; leg pain VAS 8→0; satisfaction 10/10. Returned to preoperative recreational activities, including gardening.

## Discussion

The patient presented here exemplifies the most challenging clinical scenario in spinal infection: persistent osteomyelitis with large erosive vertebral body defects, previous instrumentation, an adjacent prior burst fracture, virulent gram-negative organisms, and metabolic bone disease. Conventional teaching favours anterior debridement and corpectomy with structural cage reconstruction, supplemented by posterior instrumentation [1,2]. In this patient, however, the prior L3 burst fracture with significant endplate irregularity meant that a true anterior reconstruction would have required corpectomy at L1, L2, and L3, with anterior arthrodesis from T12 to L4. Such a procedure would have been technically demanding and likely associated with substantial morbidity, particularly in the setting of osteoporosis and prior infection.

After preoperative discussion with the patient regarding the relative merits of anterior versus posterior-only approaches, the decision was made to attempt a thorough posterior debridement with adequate transpedicular packing of synthetic graft, supported by long-segment quad-rod posterior instrumentation. CT imaging at eight months confirmed bridging bone through the L1-L2 defect with early anterior column consolidation, an encouraging result in a salvage setting.

Several advantages of this strategy are evident, namely: avoidance of anterior morbidity, as there was no anterior approach, no corpectomy cage, and no need to violate previously operated anterior tissue planes. Furthermore, there was reduced operative complexity, as a single posterior approach permitted simultaneous decompression, debridement, instrumentation, and bone grafting.

Additionally, despite a hostile, post-infectious, osteoporotic environment, early anterior column consolidation was observed by eight months with HPSBG as the sole intervertebral material [3-5]. By 12 months, repeat CT demonstrated bridging trabecular bone across the L1-L2 defect with continuity to the host endplates and no residual lucency, a notable structural recovery in a segment that had shown progressive vertebral body destruction on presentation. Thorough debridement, long-segment stabilisation, local antibiotic-loaded calcium sulfate, systemic antimicrobial therapy, and anabolic therapy with teriparatide all plausibly contributed to both infection control and bony healing. Notably, HPSBG did not cause a secondary infection, a complication that has been reported with some bone graft materials, and no recurrent infection was observed through 12 months [6, 12]. Cohesion from the graft is relevant here because the reconstruction provided no container. Comparative in vitro testing indicates that graft materials differ markedly in this respect under fluid exposure [11], though that testing was static and does not predict clinical fusion.

Posterior-only debridement and instrumented fusion has been increasingly described as an alternative to combined anterior-posterior surgery for selected patients with pyogenic spondylodiscitis, with reported success in achieving infection control and arthrodesis [1,13]. The present case extends this concept to the most demanding salvage scenario, multilevel vertebral body destruction with prior failed instrumentation, and replaces structural anterior support with a stand-alone hydrophilic synthetic graft packed transpedicularly. This differs from prior stand-alone use of the same graft, in which it was contained within a titanium corpectomy cage that supplied anterior column support [12]; here the graft was the sole intervertebral material without any cage. Reported rates of treatment failure, persistent infection, and non-union after surgical management of infected spines remain substantial across the literature [1,2,13], and the radiographic result observed here is therefore encouraging. Although limited to a single patient, this case suggests that a stand-alone hydrophilic synthetic graft such as HPSBG may be a feasible adjunct for anterior column reconstruction in carefully selected salvage settings where placement of an interbody cage is not possible or may not be optimal. Further evaluation in larger cohorts is warranted.

Mechanistic insights

Several biologic principles may plausibly contribute to graft incorporation in this setting [3-5, 7-10]. Expansion, interconnected porosity and fluid uptake are general properties of hydrophilic calcium-phosphate grafts; cohesion is a structural property for which direct comparative in vitro data exist [11]. None can be established from a single case.

Firstly, in a debridement cavity, a material that swells against the walls of the void draws blood and marrow into its interior as it is delivered and remains apposed to the bony margins once packed [11]. That apposition matters: osteoprogenitor and endothelial cells enter a scaffold across the graft-host interface, and where a graft subsides or leaves a gap, those cells must first bridge a void before ingrowth can begin [5, 7]. Sustained contact shortens the distance to be covered.

Secondly, interconnected pores give vessels and cells a path through the scaffold rather than only around it, while the surface character of the calcium-phosphate particles influences how readily osteoprogenitors attach and mature once they arrive [7-10]. These features govern the rate at which a scaffold is populated and revascularized, and they are shared across the calcium-phosphate class.

Thirdly, the web-interlaced fibre architecture combined with porous synthetic particles provides a cohesive scaffold that can be packed densely into irregular bony voids, resists migration under irrigation, and maintains graft integrity under physiological loading while enabling cellular ingress along fibre surfaces [11, 12].

Fourthly, a scaffold that takes up fluid may also permit fluid to move through it [11], raising the possibility that systemically administered antibiotic reaches the graft interior rather than tracking around a sealed mass. This remains a plausible mechanism rather than a demonstrated one, and in this patient it cannot be separated from the antibiotic-loaded calcium sulfate placed at the same sitting or from six weeks of culture-directed intravenous therapy [2].

Calcium-phosphate scaffolds with hydrophilic surfaces and submicron-scale surface topography bind matrix proteins more readily, support the attachment of marrow stromal cells, and favour their progression toward an osteoblastic phenotype [7-10]. Nanostructured calcium phosphates incorporating silicate have shown faster bone formation still in preclinical models, an effect attributed to the interaction between ionic release and surface texture [14,15]. HPSBG is built on the same chemistry: its particles are a silicate-substituted carbonate apatite rather than stoichiometric hydroxyapatite, approximating the carbonated, ion-substituted mineral phase of native bone, and this biomimetic composition is associated with more rapid resorption, remodelling and new bone formation than hydroxyapatite in preclinical models [15-17]. Suspending that chemistry within the web-interlaced fibre scaffold delivers it as a single cohesive, packable mass [11,12]. Of the properties described above, expansion and cohesion are the ones that bear on a cageless reconstruction specifically. Expansion holds the graft against the debrided bony margins rather than allowing a gap to open at the interface; cohesion keeps it within the cavity it was packed into rather than dispersing through a transpedicular corridor under irrigation [11]. A graft that both fills a void and stays in it presents a continuous framework across which vessels and osteoprogenitors can advance [5,7-9], and through which systemically delivered antibiotics may reach the interior rather than tracking around a displaced mass. Where no cage and no other structural material are placed, that framework is the whole of what the anterior column has to work with. The pairing of a bone-like surface chemistry with a scaffold that stays where it is packed is, in principle, well matched to an irregular, uncontained, infected defect of this kind; whether these properties translate into arthrodesis in the infected, metabolically compromised spine is a question for prospective comparative study.

Limitations

This is a single-patient case report, and the outcome cannot be generalised. Fusion was assessed on static computed tomography at 8 and 12 months; dynamic flexion-extension radiographs were not obtained, and a formal fusion grade was not applied. Pelvic tilt, sacral slope, sagittal vertical axis, and detailed metabolic indices were not quantified. Because debridement, fixation, antibiotic therapy, and teriparatide were applied concurrently, the independent contribution of the graft cannot be isolated.

## Conclusions

This case suggests that OsteoFlo® HydroFiber™, when packed densely into a debrided vertebral body and disc space defect and supported by long-segment posterior instrumentation, may contribute to anterior column consolidation without anterior surgery in the setting of persistent osteomyelitis, with progression from early consolidation at eight months to bridging trabecular bone at 12 months and concurrent improvement in pain and function. Because debridement, long-segment fixation, local and systemic antibiotics, and teriparatide were used concurrently, the contribution of the graft cannot be isolated in a single case. All-posterior reconstruction using a hydrophilic synthetic graft may therefore represent a reconstructive option in selected patients when anterior reconstruction or interbody cage placement is not feasible, and the alternative would otherwise be prolonged immobilisation and non-operative management, warranting prospective comparative evaluation against traditional anterior-posterior strategies.
